# Changes in phenolic profile, physicochemical properties and sensory attributes of steamed bread fortified with golden kiwifruit (*Actinidia chinensis*) flour during processing

**DOI:** 10.1002/jsfa.70535

**Published:** 2026-02-25

**Authors:** Jiecheng Li, Bai Nishran Usman Candao, Fan Zhu

**Affiliations:** ^1^ College of Food Science and Engineering, Tianjin University of Science & Technology Tianjin China; ^2^ School of Chemical Sciences, University of Auckland Auckland New Zealand; ^3^ Cotabato Sanitarium and General Hospital Sultan Kudarat Philippines

**Keywords:** Antioxidant activity, starch digestibility, vitamin C, epicatechin, procyanidin B2, steaming

## Abstract

**BACKGROUND:**

Chinese steamed bread (CSB) is a traditional East Asian staple. Its texture and nutrition depend on the gluten and starch content of the wheat flour that it contains. Incorporation of golden kiwifruit, which is rich in dietary fiber, vitamin C, and phenolic antioxidants, offers a novel fortification strategy. It may improve dough functionality and sensory attributes and provide nutritional benefits while utilizing kiwifruit waste.

**RESULTS:**

Increasing kiwifruit powder levels (0 – 500 g kg^−1^) significantly reduced protein and starch content, affecting the water absorption, mixing time, and rheology of dough. Incorporation of kiwifruit decreased pasting viscosities, starch gelatinization enthalpy and *in vitro* starch digestibility but increased phenolic content, antioxidant potential, and microbiological shelf life. Structural alterations and enzymatic activity during fermentation improved the extractability of phenolics and vitamin C, while thermal processing and storage led to degradation and reduced availability of the bioactives due to oxidation and starch retrogradation. Textural analysis revealed increased hardness and chewiness, attributed to dietary fiber and gluten network disruption, but staling rates declined. Color analysis indicated darker crumbs, aligning with sensory evaluation with lower acceptability.

**CONCLUSION:**

Kiwifruit powder fortification markedly enhanced CSB's nutritional profile and modulated dough rheology, pasting properties and thermal behavior. Although kiwifruit flour disrupted gluten network formation, leading to firmer, less elastic bread and darker crumb, it greatly prolonged freshness by slowing starch retrogradation and microbial spoilage. An incorporation level of up to 200 g kg^−1^ kiwifruit flour offered an optimal balance between functional benefits and consumer acceptability, supporting its application in traditional bakery products. © 2026 The Author(s). *Journal of the Science of Food and Agriculture* published by John Wiley & Sons Ltd on behalf of Society of Chemical Industry.

## INTRODUCTION

Chinese steamed bread (CSB) is a primary staple food in the East Asian diet. It is mainly made from wheat flour, water, and yeast or sourdough.^1^ It can be fortified with ingredients or chemical components, such as bean flours, linseeds, purple sweet potato, and red beetroot, to improve its quality and nutritional properties.^2^, ^3^, ^4^, ^5^ Microbial diversity in the sourdough used to make CSBs can influence the aroma and flavor of the final product.^6^ The production procedures of CSB include mixing the ingredients, fermentation, molding, proofing, and steaming.^1^ Fermentation is an important step that affects the rheological characteristics of the dough and the final quality of the CSB.^7^ Steaming may better retain the diverse endogenous and fortified nutrients as compared to baking.^1^ Each manufacturing step can affect the composition, color, textural attributes, sensory properties and nutritional values of CSBs, as well as related changes in phenolic composition and antioxidant capacity due to the incorporated functional ingredients.^4^, ^5^

Golden kiwifruit (*Actinidia chinensis*) is a variety of kiwifruit that originated from China.^8^ It is grown commercially and processed, along with the green kiwifruit (*A. deliciosa*), as one of the two main kiwifruit cultivars.^8^ In comparison with green kiwifruit, golden kiwifruit has more vitamin C and a higher antioxidant capacity.^9^ The total global production of kiwifruits reached approximately 4.5 million metric tonnes in 2024, the main producer countries being China, New Zealand, and Italy.^10^

Golden kiwifruit is composed primarily of starch (~500 g kg⁻¹, dry basis (db) at commercial harvest), protein (~40 g kg⁻¹, db), lipid (~20 g kg⁻¹, db), and ash (~40 g kg⁻¹, db).¹¹ It is rich in pectins, phenolic compounds (flavonoids and phenolic acids), and carotenoids.^12^, ^13^ These phenolic fractions of golden kiwifruit display antioxidant, anti‐inflammatory, antidiabetic, and other health effects.^8^ Procyanidin B2 and (‐)‐epicatechin are among the main phenolic compounds in golden kiwifruit.^14^ Vitamin C content in kiwifruit, up to 1.61 g kg^−1^ fresh mass,^9^ is higher than that of most other fruits. The peel of golden kiwifruit has higher fiber and phenolic content than the pulp and core.^13^ However, its rough texture makes it unpalatable for consumers, rendering it a waste product in processing. Seasonal limitations and unstable storage conditions further contribute to fresh kiwifruit losses for farmers. Dried kiwifruit powder is regarded as a key ingredient, and its use could be a novel way to promote sustainable use of underutilized kiwifruit.

There is growing interest in the development of functional foods. Several studies have incorporated kiwifruit starch into CSBs to make low glycemic index (*GI*) and foods that are acceptable to consumers.^15^, ^16^ However, the effects on CSBs of other functional components in kiwifruit, such as sugars, dietary fiber, and phenolic compounds, remain underexplored. Dose‐dependent effects of kiwifruit powder on starch–protein interactions and physicochemical properties of CSB dough have also not been evaluated fully.

This study aimed to: (a) quantify the retention of procyanidin B2, (‐)‐epicatechin, and vitamin C in CSBs fortified with 100 – 500 g kg^−1^ golden kiwifruit powder; (b) characterize the dose‐dependent impacts on starch gelatinization, gluten network formation, and antioxidant capacity; and (c) establish optimal incorporation levels, balancing nutritional enhancement with sensory acceptability.

It was hypothesized that the incorporated kiwifruit powder, containing high dietary fiber and phenolic contents, could significantly change the properties of the dough and correspondingly enhance the nutritional values of the CSB. Processing would alter dough structure and change the antioxidant capacity of phenolics. Fermentation and steaming may preserve phytochemical activity despite partial thermal degradation.

This study was intended to provide new findings related to the thermal behavior of kiwifruit‐derived bioactive substances in starch‐based systems. These findings would guide the development of CSBs with improved nutritional properties while maximizing phytochemical retention. Such formulations would also support the valorization of kiwifruit processing waste.

## MATERIALS AND METHODS

### Materials

Golden kiwifruits (‘Zespri’) were provided by Auckland Pack and Cool (Auckland, New Zealand). Kiwifruit powder was prepared by slicing washed, unpeeled fruits (5 mm), freeze drying, grinding to pass through a 500 μm sieve, and storing at room temperature. Wheat flour and active dried yeast (*Saccharomyces cerevisiae*) were purchased from Edmonds (Goodman Fielder Ltd., Auckland, New Zealand). (‐)‐Epicatechin (≥ 900 g kg^−1^) and l‐ascorbic acid standards were bought from Merck (Darmstadt, Germany). Procyanidin B2 (980 g kg^−1^) was obtained from AK Scientific, Inc. (Union City, CA, USA). Amyloglucosidase (200 U mL^−1^), total starch and dietary fiber assay kits and GOPOD reagent were purchased from Megazyme (Wicklow, Ireland). Invertase (≥ 300 units mg^−1^) and porcine pancreatin (3000 BPU g^−1^) were obtained from Sigma‐Aldrich (St Louis, MO, USA). All other chemicals and solvents were of analytical grade.

### Analysis of flour systems

#### Chemical composition analysis

The proximate composition (moisture, crude lipid, protein and ash), total starch, dietary fiber, and sugar profiles of the wheat flour and kiwifruit powder were determined using AACC methods^17^ (AACC 44–40.01, 30–25.01, 46–16.01, and 08–01.01), and high‐performance anion‐exchange chromatography with pulsed amperometric detection (HPAEC‐PAD), respectively. The detailed methodology and results are provided in the Supporting Information (Methods and Table S1).

#### Mixograph study

The mixing properties of flour blends were analyzed using a 10 g mixograph (National Manufacturing Co., Lincoln, NE, USA) following AACC International Approved Method 54‐40.02^17^ with slight modifications. Kiwifruit flour (KF) blends were prepared from dried powders by substituting wheat flour with kiwifruit powder at levels of 0, 100, 200, 300, 400, and 500 g kg^−1^ (designated as KF‐0, ‐100, ‐200, ‐300, ‐400, and ‐500, respectively). The preliminary mixograph absorption was initially estimated using the following regression equation:^17^

$$\text{mixograph absorption}\;\left(\%\right)=\left(\text{protein}\%\;\times \;1.5\right)+43.6$$

Due to the altered hydration properties of the flour blends, the water levels for each sample were adjusted manually in successive trials to achieve the optimum dough consistency. These experimentally determined values were recorded as the measured mixograph absorption. The mixing curves were evaluated using MixSmart software (version 2.0.609) to generate the time to peak dough resistance (mixing time).

#### Pasting analysis and gel texture after pasting

The pasting characteristics of flours were analyzed using an Anton Paar Physica MCR 301 rheometer (Graz, Austria) equipped with a starch cell and a ST24‐2D/2 V/2 V‐30 probe, as described by Li and Zhu with slight modifications.^11^ A sample slurry (3.0 g in 20 mL of MilliQ water) was subjected to a shear rate of 160 s^−1^ under a programmed heating–cooling cycle: 50 °C for 5 min, heating to 95 °C in 7.5 min, holding at 95 °C for 5 min, cooling to 50 °C in 7.5 min, and a final hold at 50 °C for 2 min. From the resulting pasting curve, the peak viscosity (PV), pasting temperature (P_temp_), peak temperature (PT), time to reach PV (P_time_), and hot paste viscosity (HPV) were recorded. Breakdown (BD = PV – HPV), final viscosity (FV), and setback (SB = FV – HPV) were calculated.

After pasting, the gel obtained from the rheological analysis was transferred to sealed vials and stored vertically at 4 °C for 24 h to set. Texture profile analysis was performed using a TA‐XT Plus texture analyzer (Stable Micro Systems, Godalming, UK) equipped with a 5 mm cylindrical probe. The gel was penetrated to a depth of 15.00 mm at a test speed of 0.5 mm s^−1^, with pre‐ and post‐test speeds of 1.0 mm s^−1^ and a 0.03 N trigger force. Hardness, adhesiveness, and springiness were determined directly from the force–time curve using Exponent software (version 5.0.6.0), and chewiness was calculated.

### Preparation of CSB

Kiwifruit Chinese steamed buns (KCSB) were prepared following the method described by Zhu and Sun^18^ with a few modifications. Various substitution levels of KF blends were used to make KCSBs, which were designated as KCSB‐0, ‐100, ‐200, ‐300, ‐400, and ‐500, respectively. The preparation process began by mixing 200 g of a flour blend with an activated yeast suspension (8 g yeast in 100 mL water at 38 °C for 10 min) in a dough kneader. The dough then underwent a first proofing (40 min, 38 °C, 80% to 85% relative humidity). After degassing by hand‐kneading for 3 min, the dough was shaped and subjected to a second proofing (for 20 min) under the same conditions. Finally, the shaped dough was steamed for 19 min. The finished buns were cooled to room temperature, wrapped, and stored at 4 °C for 24 h to stale.

To investigate physicochemical changes during processing, intermediate samples were collected at the mixing, proofing, steaming, and storage stages (as shown in Supporting Information Fig. S1). These samples were immediately freeze‐dried, ground to pass a 0.5 mm sieve, and stored in a desiccator for subsequent analysis.

### Changes of thermal properties during processing

The thermal properties of samples from each processing stage were analyzed by differential scanning calorimetry (DSC, TA Instruments Q1000, New Castle, DE, USA), following the method described by Cui and Zhu.^5^ Samples (3.5 mg, db) were hydrated with MilliQ water (1:3, *w/w*) in hermetically sealed pans for 30 min prior to analysis. They were then scanned from 25 – 90 °C at 10 °C min^−1^ against an empty reference pan. The onset (*T*_o_), peak (*T*_p_), conclusion (*T*_c_), and gelatinization enthalpy (*ΔH*) were obtained using Universal Analysis 2000 software (version 4.1D).

### Changes of color properties during processing

The color properties of CSB samples collected from each processing step were measured following the method described by Zhu and Sun.^18^ For analysis, each powder was packed into a standard Petri dish and its surface was flattened to ensure uniformity. A CR‐400 color‐meter (Minolta Camera Co., Osaka, Japan) was used to determine the CIE L* (lightness), a* (redness–greenness), and b* (yellowness–blueness) values.

### Changes of phenolic compounds during processing

Based on preliminary dose‐dependent results for the KCSB samples, three representative formulations (KCSB‐100, ‐300, and ‐500) were selected for phenolic compound analysis. These samples were chosen to reflect the observed increases in antioxidant activity and phenolic content with higher KF substitution levels during processing.

#### Extraction of phenolics

Extraction of phenolic compounds was performed following the method described by Sabaragamuwa and Perera^19^ with some modifications. Each ground CSB sample (0.50 g) was mixed with 10 mL of 90 mL L^−1^ methanol. The mixture was then centrifuged at 2500×*g* for 15 min, and the supernatant was filtered (0.45 μm cellulose‐acetate) prior to subsequent analysis.

#### Total phenol content and *in vitro* antioxidant potential

The total phenol content (TPC) and *in vitro* antioxidant potentials were determined using a 96‐well microplate reader, based on the methods described in previous studies^4^, ^19^ with a few modifications. The TPC was measured using the Folin–Ciocâlteu method. In each well, 20 μL of sample extract was mixed with 100 μL of 100 mL L^−1^ Folin–Ciocâlteu reagent. After incubating for 5 min, 80 μL of 75 g L^−1^ sodium carbonate solution was added. The mixture was incubated for 60 min at room temperature in dark conditions and absorbance was read at 765 nm. The TPC was quantified against a gallic acid standard curve and expressed as g of gallic acid equivalents (GAE) per 1 kg sample (db). For *in vitro* antioxidant potentials analysis, the 2,2‐diphenyl‐1‐picrylhydrazyl (DPPH) free radical scavenging activity assay was initiated by mixing 20 μL of sample extract with 180 μL of a 0.04 g L^−1^ methanol DPPH solution. After 30 min incubation in dark conditions at room temperature, the absorbance was measured at 517 nm. The ferric reducing antioxidant power (FRAP) was assessed using a working solution of acetate buffer (0.3 mol L^−1^, pH 3.6), 2,4,6‐tripyridyl‐*s*‐triazine (TPTZ) (0.01 mol L^−1^) and FeCl_3_ (0.02 mol L^−1^) mixed in a 10:1:1 (*v/v/v*) ratio. The reaction was initiated by mixing 20 μL of sample extract with 180 μL of the FRAP working solution. After a 10 min incubation at 37 °C, the absorbance was measured at 593 nm. For both antioxidant potential assays, activity was calculated from a Trolox standard curve and expressed as mol of Trolox equivalents (TE) per 1 kg sample (db). Methanol was used as the blank.

#### Identification and quantification of phenolic compounds in KCSB

Identification and quantification of phenolics were conducted using liquid chromatography–mass spectrometry (LC–MS) following the method described by Cui *et al*.,^4^ with some modifications. Standards of epicatechin and procyanidin B2 were prepared in 900 mL L^−1^ methanol.

Preliminary identification was performed on an Agilent 1260 Infinity II high‐performance liquid chromatography (HPLC) system (Agilent Technologies Inc., Santa Clara, CA, USA) with a diode array detector (DAD). Separation was achieved on a Poroshell 120 EC‐C18 column (100 × 4.6 mm, 4 μm) (Phenomenex, Torrance, CA, USA) using an isocratic mobile phase of 1 mL L^−1^ formic acid in water (A) and acetonitrile (B) at 0.5 mL min^−1^. The injection volume was 20 μL, with detection at 280, 320, and 380 nm.

Confirmation was conducted on an Agilent 6460 triple quadrupole LC–MS system with an electrospray ionization (ESI) source in negative mode. Separation used a Zorbax Eclipse RRHD column (5 × 2.1 mm, 1.8 μm, Agilent Technologies Inc.) with a gradient elution of solvents A and B at 0.35 mL min^−1^. Epicatechin and procyanidin B2 were confirmed by comparing their retention times and mass spectra (*m/z* 289 for epicatechin, *m/z* 577 for procyanidin B2) with those of the standards. Data were processed using MassHunter Qualitative Analysis software (version B.07.00, Agilent Technologies Inc.).

Quantification was performed using the HPLC‐DAD method described above. Calibration curves were generated using serial dilutions of epicatechin and procyanidin B2 standards (6.25 – 100 mg L^−1^). Compound concentrations were calculated from the peak areas at the corresponding retention times. The quantification data were cross‐validated with LC–MS results to ensure accuracy.

#### Identification and quantification of vitamin C

Identification and quantification of vitamin C were carried out using a Hewlett Packard 1100 HPLC system (Livonia, MI, USA) equipped with a DAD. Chromatographic separation was achieved on a Phenomenex Synergi MAX‐RP column (250 × 4.6 mm, 4 μm) (Phenomenex) with an isocratic mobile phase of 1 mL L^−1^ formic acid and methanol (25:75, *v/v*) at a flow rate of 1.0 mL min^−1^. The injection volume was 20 μL and the detection wavelength was 254 nm. Quantification was based on a calibration curve generated from vitamin C standards (1, 5, 10, 25, and 50 μg mL^−1^) prepared in Milli‐Q water.

### Analysis of CSB quality

#### Moisture content and water activity

The moisture content of KCSB was determined using a moisture meter (Infrared MA35, Sartorius, Göttingen, Germany), and the water activity (a_w_) was measured using a water activity meter (AquaLab 4 TE, Pullman, WA, USA).

#### Color analysis

The color properties (L*, a*, and b*) of the fresh CSB were measured using the same procedure described for the CSB samples collected from processing steps. For each fresh CSB sample, measurements were taken at three randomly selected locations on both the crust and crumb. From the L*, a*, and b* values, the whiteness index was calculated using the following equation:

$$\text{whiteness index}=100-\sqrt{{\left(100-L^{*}\right)}^{2}+{\left(a^{*}\right)}^{2}+{\left(b^{*}\right)}^{2}}$$

#### Volumetric properties

The volumetric properties of fresh CSB were evaluated using a VolScan analyzer (VSP 600, Stable Micro Systems).^18^ Volume, maximum width, and maximum height of CSB were obtained from the VolScan software (version 1.0.3.0). Specific volume was calculated as the ratio of the volume to the weight of CSB. Spread ratio was determined as the ratio of the maximum width to the maximum height of CSB.

#### Textural analysis

The textural attributes of CSB were obtained using a TA‐XT texture analyzer (Stable Micro Systems) with a two‐bite texture profile analysis (TPA), following the method described by Zhu and Li.^3^ A 35 mm cylindrical probe was used to compress the sample 25 mm at a test speed of 10.0 mm s^−1^ (pre‐ and post‐test speeds: 5.0 mm s^−1^) with a 5.0 g trigger force. Hardness, cohesiveness, springiness, and chewiness were determined from the resulting force–time curve, using Exponent software (version 5.0.6.0). The staling rate (%) was calculated as the percentage increase in hardness during storage divided by the initial hardness of the fresh CSB.

#### *In vitro* starch digestibility and expected glycemic index

The *in vitro* starch digestibility and *eGI* of freshly cooked CSB were determined based on the methods described by Englyst *et al*.^20^ and Goñi *et al*.^21^ with some modifications. Samples (1.0 g) were slurried with 5 mL of water. Starch hydrolysis was initiated by adding a multi‐enzyme solution containing pepsin, pancreatin, amyloglucosidase, and invertase in 0.1 mol L^−1^ sodium acetate buffer, pH 5.2, and incubating the mixture in a shaking water bath at 37 °C for 120 min. Aliquots (0.1 mL) were taken at 20 min intervals, and the enzymatic reaction was immediately quenched with 800 mL L^−1^ ethanol (2 mL). The glucose content in the supernatant was then quantified using a glucose oxidase‐peroxidase (GOPOD) kit. Starch content was calculated by multiplying the measured glucose content by a factor of 0.9. The rapidly digestible starch (RDS), slowly digestible starch (SDS), and resistant starch (RS) fractions were determined based on the hydrolysis curve. Total starch content for flour mixtures was determined proportionally based on the substitution ratio of wheat flour and KF powder. A first‐order kinetic model was fitted to the data, and the area under the curve (AUC) was calculated using Origin software (version 9.0, OriginLab Corporation, Northampton, MA, USA). The hydrolysis index (HI) was determined as the ratio of the sample's AUC to that of a white bread reference. The *eGI* was calculated as described by Goñi *et al*.^21^

#### Microbiological shelf‐life analysis of CSB

The microbiological shelf life of CSB was evaluated by monitoring mold growth, following a modified method described by Zhu and Sun.^18^ Fresh CSB samples were aseptically sliced (20 mm thickness). The slices were individually packaged in partially sealed plastic bags to maintain aerobic conditions and stored at 20 °C. Samples were monitored daily for 7 days, and shelf life was defined as the first day on which mold growth was seen.

#### Sensory evaluation

Sensory evaluation of fresh CSB was conducted following the method described by Cui *et al*.^4^ with slight modifications. A trained panel of 31 participants from the University of Auckland evaluated the descriptive profile and overall acceptability of the samples. The evaluation was performed in individual sensory compartments, where panelists received six types of uniform CSB pieces presented simultaneously under randomized codes. Panelists scored the samples for whiteness, smoothness, hardness, elasticity, cohesiveness, fruity aroma, stickiness, chewiness, sweetness, sourness, and overall acceptability (Supporting Information file). The study was approved by the University of Auckland human participants ethics committee (reference number 24758).

### Statistical analysis

All measurements were carried out in triplicate on three independent samples for each group. Statistical analysis was conducted using one‐way analysis of variance (ANOVA), with significant differences determined by Duncan's multiple range test at a significance level of *P* < 0.05. Data were analyzed using SPSS Statistics (version 23.0; IBM Corporation, Armonk, NY, USA).

## RESULTS AND DISCUSSION

### Mixing properties

The protein content of CSB decreased significantly with an increase in the KF content, from 133.5 g kg^−1^ of KCSB‐0 to 94.6 g kg^−1^ of KCSB‐500 (Table 1). This reduction was due to the lower protein content of KF (55.6 g kg^−1^) in comparison with wheat flour (134 g kg^−1^). The mixograph absorption declined strongly with increasing KF content (Table 1). This was attributed to the high insoluble dietary fiber content (123 g kg^−1^) and soluble sugar content (~ 495 g kg^−1^) in KF, which reduced the gluten‐forming proteins, increased the initial dough consistency, and reduced the amount of water required to reach the target dough resistance.^22^ Insoluble fiber diluted the gluten network and increased dough stiffness, whereas soluble sugars competed with gluten proteins for water, limiting gluten hydration. Less water was therefore needed during mixing to reach peak dough resistance, leading to lower absorption values. The reduction in the mixing time was primarily due to the lower gluten content in KF in comparison with wheat flour. The decreased gluten content reduced the capacity for gluten network formation, thus requiring less hydration and a shorter mixing time. These findings were consistent with previous studies.^4^, ^5^ Previous studies also reported that phenolic acids (e.g., caffeic acid and chlorogenic acid) in KF could interact with gluten proteins and disrupt disulfide bonds, weakening the dough structure.^22^ However, in this study, the impact of phenolics on mixing behavior was likely supplementary rather than a primary factor, with gluten dilution being the dominant factor.

**Table 1:** Mixing, pasting, and dynamic oscillatory properties of kiwifruit flour and wheat flour mixtures

Samples	Moisture (g kg^−1^)	Protein (g kg^−1^)	Measured mixograph absorption (%)	Mixing time (min)	PV (Pa s)	HPV (Pa s)	FV (Pa s)	Breakdown (Pa s)	Setback (Pa s)
KF‐0	125.5 a	133.5 a	60.10 a	4.85 a	2.45 a	1.08 a	2.28 a	1.37 a	1.21 a
KF‐100	126.1 a	125.7 b	59.17 b	2.66 b	2.26 b	0.94 b	2.07 b	1.33 a	1.12 a
KF‐200	126.6 a	117.9 c	57.87 c	2.16 b	2.14 c	0.81 c	1.76 c	1.33 a	0.95 b
KF‐300	125.7 a	110.1 d	57.27 c	1.05 c	1.51 d	0.69 d	1.40 d	0.82 b	0.71 c
KF‐400	127.7 a	102.4 e	56.37 d	0.95 c	1.07 e	0.51 e	0.96 e	0.56 c	0.45 d
KF‐500	128.2 a	94.6 f	55.47 e	0.95 c	0.69 f	0.36 f	0.68 f	0.33 d	0.32 e

KF, kiwifruit flour; Measured mixograph absorption (%), values were determined experimentally to reach optimum consistency; PV, peak viscosity; HPV, hot paste viscosity; FV, final viscosity. KF‐0–500, a range of flour mixtures where kiwifruit flour replaces 0 to 500 g kg^−1^ of wheat flour by weight. Different letters in the same column indicate significant differences (*P* < 0.05). The experiment was performed in triplicate.

### Pasting properties and gel texture after pasting

Peak viscosity exhibited a decreasing trend as the KF content increased, dropping from 2.45 Pa s at KF‐0 to 0.69 Pa s at KF‐500 (Table 1). This decline was attributed primarily to starch and gluten dilution and the high water‐binding capacity of KF's dietary fibers, which restricted starch granule swelling and breakdown.^5^ The high sugar content (~ 495 g kg^−1^) in KF, particularly glucose and fructose, also hindered starch gelatinization by competing for water and forming hydrogen‐bonded starch‐sugar complexes.^5^, ^11^ Pectin in KF might form a thin ‘film’ around starch granules, limiting amylose leaching. The acidic environment also promoted partial starch hydrolysis, releasing glucan polymers that contributed to residual viscosity.^11^ The decline in hot paste viscosity (HPV) indicated reduced thermal stability, likely due to shear‐induced disruption of starch granules and the presence of hemicellulose and cellulose enhancing this breakdown. Final viscosity (FV) also decreased with KF addition, as sugars, pectin, and phenolics inhibited amylose–amylose reassociation during cooling through hydrogen bonding, resulting in weaker gel reformation.^11^

After pasting, the hardness, adhesiveness, springiness, and chewiness of the resulting gels all declined (Supporting Information, Table S2). The softer texture was attributed to KF's sugars and soluble (pectic, 36.2 g kg^−1^) and insoluble (cellulosic and hemicellulosic, 123 g kg^−1^) fibers interfering with starch retrogradation and the cross‐linking of amylose chains.^18^ Gluten dilution further weakened the gel matrix, reducing its elasticity and cohesiveness.^5^ Although dietary fibers could form entangled polymeric chains within the gel network, their interaction with starch and sugars disrupted gel compactness, producing a softer and less resilient structure.

### Thermal properties of KCSB samples collected from processing steps

The gelatinization temperatures (*T*_o_, *T*_p_ and *T*_c_) of CSB samples were increased with the higher KF content (Table 2). This was attributed to the high sugar content in KF. The hydrophilic sugars decreased water availability in the flour system and reduced the interactions between water and starch molecules. Sugars induced the intermolecular interactions with starch by hydrogen bonds, contributing to the increased gelatinization temperatures.^23^ No significant changes in gelatinization temperatures were observed across different stages of CSB processing, including mixing, fermentation and proofing. However, after freezing storage for 1 week, both the gelatinization temperatures and enthalpy changes showed a marked decrease. This suggested that the granular structure of starch was modified during freezing. The decline in *ΔH* with increasing KF content was likely due to the reduction in starch concentration as higher amounts of KF were incorporated into the CSB formulation. Phenolic compounds in the KF might interact with other dough components during storage, limiting starch gelatinization and contributing to the observed decrease in Δ*H*.^4^ The monosaccharides (mainly glucose and fructose) in KF had little impact on the Δ*H*.^23^ The decreased Δ*H* suggested that the addition of KF to CSB led to a disruption in molecular order during gelatinization. No consistent trends in Δ*H* were observed from mixing to proofing, indicating that the molecular structure of CSB remained largely unchanged before cooking. Overall, alterations in the starch granular structure occurred primarily during or after cooking and storage.

**Table 2:** Thermal properties of samples collected during CSB processing

Samples	*T*_o_ (°C)	*T*_p_ (°C)	*T*_c_ (°C)	Δ*H* (J g^−1^)
KCSB‐0				
Mixing	54.8 a	60.3 a	67.9 a	4.8 b
Fermentation	53.8 a	60.1 a	68.2 a	5.9 a
Proofing	54.5 a	60.2 a	68.0 a	6.1 a
Steaming	n.d.	n.d.	n.d.	n.d.
Storage	39.4 b	43.4 b	57.0 b	3.1 c
KCSB‐100				
Mixing	59.4 a	65.0 a	74.5 a	4.6 a
Fermentation	59.9 a	64.8 a	71.2 b	4.5 a
Proofing	60.0 a	64.8 a	72.6 ab	3.9 a
Steaming	n.d.	n.d.	n.d.	n.d.
Storage	41.5 b	45.4 b	51.5 c	3.1 a
KCSB‐200				
Mixing	56.4 a	61.9 a	70.0 a	4.7 a
Fermentation	56.5 a	62.2 a	70.7 a	4.1 a
Proofing	56.7 a	61.9 a	71.4 a	4.4 a
Steaming	n.d.	n.d.	n.d.	n.d.
Storage	41.5 b	50.8 b	56.2 b	3.3 b
KCSB‐300				
Mixing	58.3 a	65.7 a	73.8 a	4.0 b
Fermentation	59.2 a	63.0 a	73.5 a	6.9 a
Proofing	59.9 a	65.6 a	73.4 a	3.9 b
Steaming	n.d.	n.d.	n.d.	n.d.
Storage	38.6 b	46.1 b	59.1 b	3.6 b
KCSB‐400				
Mixing	62.0 a	66.6 a	74.1 a	2.7 a
Fermentation	61.0 ab	66.6 a	74.7 a	2.9 a
Proofing	60.6 b	66.4 a	74.3 a	2.8 a
Steaming	n.d.	n.d.	n.d.	n.d.
Storage	41.8 c	43.6 b	49.8 b	1.7 b
KCSB‐500				
Mixing	58.7 a	63.5 a	71.3 a	2.9 a
Fermentation	58.9 a	63.5 a	71.1 a	2.5 a
Proofing	58.2 a	63.6 a	70.8 a	2.6 a
Steaming	n.d.	n.d.	n.d.	n.d.
Storage	39.6 b	45.1 b	56.1 b	1.5 b

*T*_o_, onset temperature; *T*_p_, peak temperature; *T*_c_, conclusion temperature; Δ*H*, enthalpy change; KCSB‐0 – 500, Chinese steamed bread made with wheat flour partially replaced by kiwifruit powder (0 – 500 g kg^−1^). n.d., not detected. Different letters in the same column and for the same CSB indicate significant differences (*P* < 0.05). The experiment was performed in triplicate.

### Color analysis of KCSB samples collected from processing steps

The color of the KCSB was significantly impacted by KF addition throughout processing (Supporting Information, Table S3). After mixing, KF addition decreased lightness (L*) and increased the yellowness (b*), resulting in a darker and more yellowish product. Fermentation further decreased L* values and shifted a* values positively (a redder hue) at higher KF concentrations. Proofing and steaming processes markedly reduced the L* of the KCSB while increasing both the a* and b* values, leading to an even darker and more reddish‐yellow appearance. After storage, there was a slight recovery in the lightness (L*), and a decrease in both the a* and b* values, suggesting a partial reversal in color changes. These observations reflected the impact of KF on the color changes of KCSB, which determined the final appearance of fresh KCSB.

### Changes of phenolic compounds and vitamin C during KCSB processing

Total phenol content, *in vitro* antioxidant potentials (FRAP and DPPH), and vitamin C demonstrated a dose‐dependent increase with the addition of KF (Table 3). However, the stability and extractability of these bioactive compounds were strongly influenced by the processing stage.

**Table 3:** TPC, *in vitro* antioxidant potentials and vitamin C, (‐)‐epicatechin and procyanidin B2 content of samples collected during Chinese steamed bread (CSB) processing

Samples		TPC (g GAE kg^−1^ db)	FRAP (mol TE kg^−1^ db)	DPPH (mol TE kg^−1^ db)	Vitamin C (g kg^−1^ db)	(‐)‐Epicatechin (g kg^−1^ db)	Procyanidin B2 (g kg^−1^ db)
Pure kiwifruit powder		3.5275	0.0591	0.0796	9.79	0.653	0.940
KCSB‐100	Mixing	0.984 e	0.0219 d	0.0456 b	0.644 c	0.0631 ab	0.0701 b
	Fermentation	1.68 a	0.0281 b	0.0501 a	0.776 ab	0.0643 ab	0.0933 a
	Proofing	1.53 b	0.0343 a	0.0471 b	0.812 a	0.0632 ab	0.0990 a
	Steaming	1.10 d	0.0225 c	0.0432 b	0.754 b	0.0542 b	0.0811 b
	Storage	1.19 c	0.0219 d	0.0475 b	0.676 c	0.0520 b	0.0612 c
KCSB‐300	Mixing	1.61 c	0.0297 c	0.0569 b	1.29 b	0.169 b	0.218 b
	Fermentation	2.20 a	0.0335 b	0.0538 bc	1.31 ab	0.197 a	0.232 a
	Proofing	2.12 b	0.0397 a	0.0628 a	1.75 a	0.167 b	0.215 b
	Steaming	1.39 e	0.0285 d	0.0497 c	1.50 ab	0.169 b	0.219 b
	Storage	1.58 d	0.0273 e	0.0532 bc	1.35 ab	0.141 c	0.160 c
KCSB‐500	Mixing	1.92 c	0.0392 c	0.0601 c	2.57 a	0.272 a	0.326 b
	Fermentation	2.49 b	0.0431 b	0.0755 a	2.34 bc	0.282 a	0.386 a
	Proofing	2.62 a	0.0493 a	0.0682 b	2.54 a	0.246 b	0.334 b
	Steaming	1.66 e	0.0348 e	0.0601 c	2.41 ab	0.232 bc	0.263 c
	Storage	1.73 d	0.0369 d	0.0643 c	2.23 c	0.225 c	0.250 c

TPC, total phenolic content; FRAP, ferric reducing ability of plasma; DPPH, 2,2‐diphenyl‐1‐picrylhydrazil free radical scavenging, GAE, gallic acid equivalents; TE, Trolox equivalents; db, dry basis; KCSB‐100, ‐300, ‐500, CSB made with wheat flour partially replaced by kiwifruit powder (100, 300, 500 g kg^−1^). Different letters in the same column and for the same CSB indicate significant differences (*P* < 0.05). The experiment was performed in triplicate.

During mixing, the increases in TPC, antioxidant potential, and vitamin C were not proportional to the KF levels, likely due to non‐covalent interactions between phenolics and various components (starch, sugars, and gluten) that reduced extractability.^24^, ^25^, ^26^ Changes in vitamin C (ascorbic acid) were due to the oxidative degradation of ascorbic to dehydroascorbic acid in the presence of oxygen and water^27^ and the binding of soluble ascorbic acid to the insoluble fiber matrix. Liquid chromatography–mass spectrometry analysis confirmed that the dimeric procyanidin B2 remained stable with concentrations increasing proportionally (0.070 – 0.326 g kg^−1^ db) whereas the main phenolic (‐)‐epicatechin was susceptible to oxidation and complex formation.^14^, ^28^, ^29^

Fermentation and proofing resulted in a slight increase in TPC and antioxidant potential of all samples. This correlated with the enhanced extractability of vitamin C. Long‐term fermentation induced structural alterations such as dough expansion, rearrangement of the protein–starch network. These changes might have disrupted polyphenol–dietary fiber complexes, releasing bound polyphenols and vitamin C.^30^ Similarly, Tian *et al*.^31^ found improved antioxidant activity in breads fortified with phenolic acids after fermentation.

Steaming led to a reduction in TPC and antioxidant potentials in all samples, due to the thermal degradation, as reported in previous studies.^32^ This was reflected in minor loss of vitamin C, limited by the short cooking time^1^ and a slight reduction in the concentrations of (‐)‐epicatechin and procyanidin B2, which was also attributed to the formation of sugar‐phenolic complexes that reduced extractability.

After a 7 day storage at 4 °C, vitamin C content declined significantly due to oxidation.^27^ However, a slight increase in TPC and antioxidant potential was observed. This improvement might be attributed to starch retrogradation and recrystallization disrupting phenolic complexes and enhancing their extractability.^5^ However, the specific compounds (‐)‐epicatechin and procyanidin B2 decreased slightly in the KCSB‐300 but remained stable in the KCSB‐500. This suggested that the KCSB‐500 provided a protective environment, likely due to its higher total antioxidant load and lower pH. This environment effectively shielded the (‐)‐epicatechin and procyanidin B2 from oxidative degradation and retrogradation‐induced entrapment.^28^, ^29^

### Moisture content and water activity of fresh KCSB

An increase in the proportion of KF in KCSB was correlated with a declining trend in moisture content from 423 g kg^−1^ of KCSB‐0 to 282 g kg^−1^ of KCSB‐500 (Table 4). This reduction was attributed to the negative impact of dietary fibers and other constituents of KF on gluten network formation, which in turn decreased the water holding capacity of the dough and the resulting CSB.^33^

**Table 4:** Physicochemical characteristics of fresh KCSB

Samples	Specific volume (mL g^−1^)	Spread ratio	Moisture content (g kg^−1^)	Water activity	Fresh KCSB				Staled KCSB			RDS (g kg^−1^)	SDS (g kg^−1^)	RS (g kg^−1^)	*HI*	*eGI*
					Hardness (g)	Chewiness (g)	Springiness	Cohesiveness	Staling rate (%)	Hardness (g)	Chewiness (g)					
KCSB‐0	2.12 a	1.15 a	423.1 a	0.976 a	1574 f	1841 f	0.819 a	1.16 c	5.79 a	10675 d	10775 d	247 a	133 a	434 a	100 a	94.6 a
KCSB‐100	1.20 b	1.02 a	370.5 b	0.949 b	3101 e	4153 e	0.749 b	1.34 b	2.55 b	10996 d	14708 c	219 ab	103 a	411 ab	82.7 b	85.1 b
KCSB‐200	0.87 c	0.98 a	334.1 c	0.919 c	5029 d	7457 d	0.678 b	1.52 a	1.45 c	12302 c	15600 c	174 bcd	116 a	337 bc	82.0 b	84.8 b
KCSB‐300	0.84 c	0.98 a	333.3 c	0.909 d	6782 c	11807 c	0.704 b	1.60 a	1.65 c	18391 b	21463 b	183 bc	53.0 b	319 c	79.4 bc	83.3 bc
KCSB‐400	0.83 c	0.97 a	328.9 c	0.885 e	11887 b	15006 b	0.667 b	1.55 a	0.99 d	23642 a	37086 a	148 cd	50.0 b	300 c	72.3 c	79.4 c
KCSB‐500	0.82 c	0.98 a	282.4 d	0.823 f	13997 a	21954 a	0.668 b	1.57 a	n.d.	n.d.	n.d.	133 d	27.0 b	259 c	59.8 d	72.5 d

KCSB, Chinese steamed bread fortified with kiwifruit flour. RDS, rapidly digestible starch; SDS, slowly digestible starch; RS, resistant starch; *HI*, hydrolysis index; *eGI*, expected glycemic index. KCSB‐0 – 500, Chinese steamed bread made with wheat flour partially replaced by kiwifruit powder (0 – 500 g kg^−1^). n.d., not detected. Different letters in the same column indicate significant differences (*P* < 0.05). The experiment was performed in triplicate.

Water activity decreased from 0.976 of KCSB‐0 to 0.823 of KCSB‐500 with the increasing KF content. The reduction in *a*_*w*_ could have been influenced by the dietary fiber, sugars and phenolic compounds in KF. These components could have bound with water, resulting in the reduced availability of water.^23^, ^34^ A lower *a*_*w*_ contributed to the inhibition of microbial growth and the shelf‐life extension of KCSB. An excessively low *a*_*w*_ could potentially compromise the texture and palatability of the KCSB.

### Color analysis of fresh KCSB

Increasing KF content in KCSB significantly decreased the lightness (L*) and the whiteness index of both the crust and the crumb (Supporting Information, Table S4). An increase in the red–green (a*) value and a decrease in the yellow–blue (b*) value indicated that the incorporation of KF enhanced the red hue while decreasing the yellow hue, leading to a darker appearance with a brownish hue. The overall reduction in the whiteness index further confirmed the departure of the KCSB samples from a pure white appearance as KF concentration increased. These color changes were mainly attributed to the presence of carotenoids, which provided the yellow and orange hues in kiwifruit and lowered the whiteness index. The high sugar content resulted in a brown hue due to oxidation.^35^ The difference in color between the KCSB‐0 and ‐100 was not significant. These observations were consistent with the results of the sensory evaluation. The visual perception of color was a critical factor influencing consumer acceptance, and the changes induced by KF could potentially affect the sensory attributes of KCSB negatively.

### Volumetric properties

The specific volume of fresh KCSB samples decreased significantly from 2.12 mL g^−1^ of KCSB‐0 to 0.82 mL g^−1^ of KCSB‐500 (Table 4), and the spread ratio showed a slight downward trend. The reduced specific volume suggests that the incorporation of KF adversely affected the expansion capacity of KCSBs. This effect was associated with the high insoluble fiber and sugar content in KF, which restricted gas expansion during proofing and baking.^36^, ^37^ The absence of gluten proteins weakened the dough strength and viscoelasticity, facilitating CO_2_ escaping during fermentation.^36^ Moreover, high sugar content in KF inhibited the activity of yeast and increased the osmotic pressure, further reducing gas generation and retention. These effects led to a denser dough and lower specific volume.^37^

No significant changes in specific volume and spread ratio were observed when the KF substitution levels exceeded 20%. This might be due to the acidic environment during fermentation, which promoted gluten proteolysis and increased solubility. These changes facilitated the formation of stable and extensible protein networks, which enhanced the dough's extensibility.^36^ Vitamin C (ascorbic acid) in KF could be oxidized to dehydroascorbic acid, which oxidized the native glutathione in flour, preserved disulfide bonds and minimized the SH/SS interchange reactions. This resulted in a uniform, cohesive crumb structure and improved dough elasticity, helping to preserve specific volume even at a high KF content.^30^

### Textural analysis of fresh and staled KCSB

The hardness of KCSB increased significantly from 1574 g (KCSB‐0) to 13997 g (KCSB‐500) (Table 4), indicating that KF disrupted the gluten network structure, reducing dough elasticity and resulting in denser and firmer products. The high dietary fiber content in KF bound water and restricted the hydration and swelling of starch granules,^36^ as observed in the pasting properties (e.g., reduced peak viscosity in Table 1). Pectin in KF might also form a ‘film’ around starch granules, further limiting their expansion and contributing to the increased hardness.^11^ Similarly, chewiness increased markedly from 1841 g at KCSB‐0 to 21954 g at KCSB‐500, as fiber‐starch‐gluten interactions formed a compact matrix with higher chewing resistance. The addition of KF decreased the springiness of fresh KCSB samples, due to the less gas formation/holding in the presence of insoluble dietary fiber of KF. These results were consistent with the pasting gel's springiness results (Supporting Information, Table S2). Cohesiveness increased from 1.16 (KCSB‐0) to 1.60 at KCSB‐300 and remained stable at higher substitution levels, likely due to the presence of polysaccharides (dietary fiber and pectin), improving crosslinking within the dough. This was supported by the pasting results (Table 1), which showed that the presence of dietary fibers disrupted starch granule swelling while promoting adhesive interactions in the dough.

During the extended storage, the hardness of staled samples increased notably, from 10675 g (KCSB‐0) to 23642 g (KCSB‐400). This was likely due to the starch retrogradation and moisture redistribution.^38^ The high fiber content in KF might accelerate this phenomenon by binding water, leading to a firmer structure in the staled samples. However, the staling rate decreased significantly from 5.79% (KCSB‐0) to 0.99% (KCSB‐400), aligning with reduced FV and Δ*H* during gelatinization (Tables 1 and 2). The phenolic compounds and sugars in KF likely inhibited starch retrogradation and CSB staling through interactions among starch, dietary fiber, sugars, and phenolic compounds,^4^ which stabilized the amorphous starch regions and delayed recrystallization.

### *In vitro* starch digestibility and expected glycemic index

Higher levels of KF substitution in wheat flour strongly reduced the *in vitro* starch digestibility and expected glycemic index (*eGI*) values in a dose‐dependent manner (Table 4). This effect was due primarily to the dilution of the total starch content in the system, as KF contains approximately 50 g kg^−1^ starch (Supporting Information, Table S1). The dietary fibers in KF (e.g., pectin) would encapsulate the starch granules and hinder enzymatic hydrolysis, reducing the RDS and SDS.^5^, ^11^ The high dietary fiber content and enhanced water‐binding capacity of KF may also limit starch gelatinization during steaming, reducing the accessibility of starch granules to enzymatic hydrolysis and further lowering RDS and SDS.^15^

Phenolic compounds in KF might inhibit digestive enzymes and form complexes with starch, contributing to slower starch digestion and a reduced glycemic response.^39^ These findings are consistent with those reported by Cui *et al*.^4^ for CSB fortified with red beetroot.

The lower *eGI* values for the KCSB with higher KF substitution were partly attributable to the high fructose content (257 g kg^−1^, shown in Supporting Information, Table S1). Fructose does not contribute to *eGI*, which was indicated by glucose. Incorporation of KF therefore efficiently reduced the *eGI* of CSB, suggesting its potential application in modulating starch digestibility and developing functional low‐glycemic foods for metabolic health management.

### Microbiological shelf‐life analysis

The incorporation of KF significantly extended the microbiological shelf life of CSB (Supporting Information, Table S5), a product typically spoiled by microorganisms like molds and yeasts.^38^, ^40^ KCSB‐0, ‐100 and ‐200 showed visible mold growth by day 3, associated with their higher moisture content and *a*_*w*_. In contrast, KCSB‐300, ‐400 and ‐500 remained mold free for up to 6 days. These results were consistent with previous studies on microbiological stability in linseed‐fortified CSBs.^3^ The increased shelf life might be due to the lower *a*_*w*_ and the antifungal properties of phenolics from kiwifruit powder such as procyanidin B2 and (‐)‐epicatechin, at higher inclusion levels.^40^, ^41^

### Sensory evaluation

**Figure 1:**
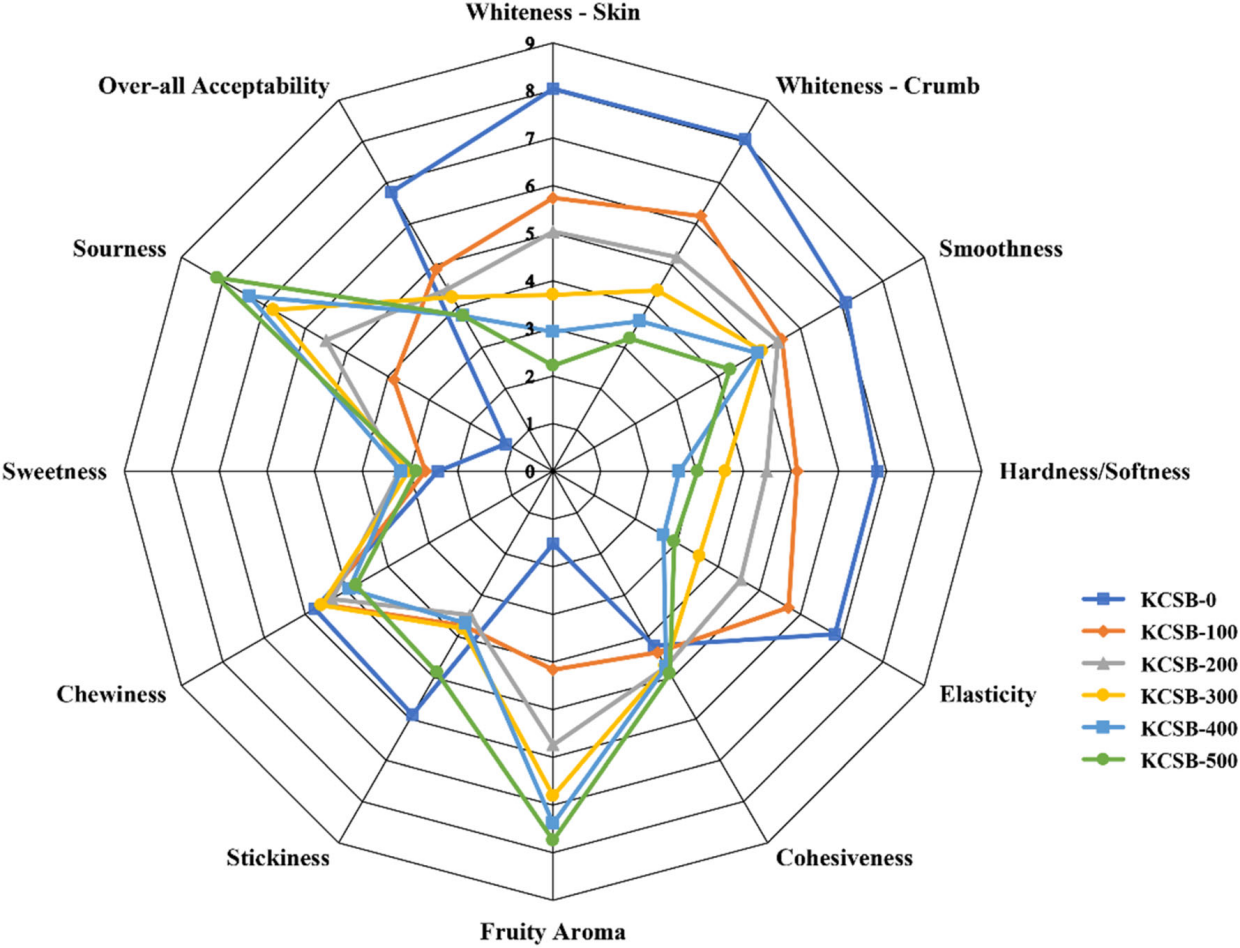
Radar chart of sensory evaluation for fresh Chinese steamed bread fortified with kiwifruit flour (KCSB).

The addition of KF significantly influenced the sensory attributes of CSB (Fig. 1). As KF substitution increased, the whiteness of CSB decreased, which lowered appearance acceptability, a critical factor for CSB, as Huang and Miskelly reported.^42^ Kiwifruit Chinese steamed buns also exhibited a stronger fruity aroma and pronounced sourness, reflecting the acidity of kiwifruit (pH 3.3 – 3.6).^11^ There was high sugar content in kiwifruit but no significant differences in sweetness were detected, as the strongly perceived sourness from the unripened kiwifruit used in this study likely masked the sweetness.

Texturally, higher KF levels produced a harder and less elastic CSB than KCSB‐0, which was attributed to gluten dilution^24^ and impaired gas retention during fermentation and steaming.^18^ Stickiness, cohesiveness, and chewiness showed no significant changes. As a result, overall acceptability scores fell with increasing KF levels, primarily due to the intensified sourness and hardness. However, health‐conscious consumers might perceive these attributes differently. Future research may focus on optimizing KF incorporation levels and processing techniques to mitigate these sensory drawbacks while preserving health benefits.

### Research significance and novelty

This study presented an innovative approach to food formulation by transforming low‐value food waste into a high‐value functional ingredient and creating new opportunities for farmers and food manufacturers. Golden kiwifruit by‐products, including imperfect fruits, undersized fruits, and surplus production fruits, are often discarded, leading to increased costs and environmental concerns. The study used the freeze‐dried golden kiwifruit powder from the whole fruit (including the peel) as a valuable ingredient to optimize resource utilization, enhance sustainability, and improve the nutritional value of the final product.

The addition of kiwifruit powder enriched KCSB with vitamin C, phenolic antioxidants, dietary fiber, and minerals, offering health benefits such as improved digestion and a lower glycemic index. This research also provided novel insights into the dynamic changes in phenolic compounds during bread processing, including mixing, fermentation, proofing, steaming, and storage, indicating the retention and transformation of bioactive compounds. The results suggested that there was potential for expanding this sustainable approach to other staple foods, benefiting producers, food manufacturers, and consumers. Future research could explore the effects of ripened and highly perishable fruits with higher starch content on the processing properties and phenolic composition of steamed bread, further broadening the application of underutilized crops in functional food development.

## CONCLUSIONS

Kiwifruit flour acted as a functional ingredient in CSB, providing nutritional benefits while modifying structural and stability properties. Interactions among KF‐derived dietary fibers, polyphenols, sugars, and starch disrupted the gluten network and starch retrogradation, resulting in altered textural profiles but improved resistance to staling. Incorporation of KF improved vitamin C extractability due to fermentation‐induced fiber degradation and pH reduction, whereas the stability of phenolic compounds varied with processing conditions, with procyanidin B2 showing greater resistance to oxidation than (‐)‐epicatechin. Interactions with starch and the dynamic changes during mixing, proofing, and steaming influenced the retention and extractability of key bioactive compounds in KF‐containing formulations. Kiwifruit flour also reduced starch digestibility, consistent with demands for low‐glycemic healthy foods. However, the balance between nutritional fortification and sensory quality, particularly increased sourness and reduced elasticity, highlights the challenges in consumer acceptance. These results illustrate KF's potential as an ingredient for innovative bakery products, offering functional benefits while maintaining acceptable sensory attributes.

## FUNDING INFORMATION

Bai Nishran Usman Candao was supported by a Manaaki New Zealand Scholarship through the New Zealand aid program.

## CONFLICT OF INTEREST

The authors declare no conflict of interest.

## Supporting information


**Data S1:** Supporting Information

## Data Availability

The data that support the findings of this study are available upon reasonable request.
