# Supplementary material for: Changes in phenolic profile, physicochemical properties and sensory attributes of steamed bread fortified with golden kiwifruit (Actinidia chinensis) flour during processing
Source: J Sci Food Agric. 2026 Feb 25;106(8):4576–86. doi: 10.1002/jsfa.70535 (PMC13157245; doi:10.1002/jsfa.70535)
Supplement: Supplementary file 1 — Data S1: Supporting Information [file JSFA-106-4576-s001.docx]

**Supplementary materials**

**Changes in phenolic profile, physicochemical properties and sensory attributes of steamed bread fortified with golden kiwifruit (*Actinidia chinensis*) flour during processing**

Jiecheng Li^1^, Bai Nishran Usman Candao^2,3^, Fan Zhu^2,*^

^1.^ College of Food Science and Engineering, Tianjin University of Science & Technology, Tianjin 300457, China

^2.^ School of Chemical Sciences, University of Auckland, Private Bag 92019, Auckland 1142, New Zealand

^3.^ Cotabato Sanitarium and General Hospital, Sultan Kudarat, Philippines

^*^ Correspondence, email: fzhu5@yahoo.com

**Supplementary methods**

*Chemical composition analysis*

The proximate composition (moisture, crude lipid and ash) of kiwifruit powder was determined using the AACC methods^17^ of 44-40.01, 30-25.01, 46-16.01 and 08-01.01, respectively. Total starch and total dietary fibre contents of both wheat flour and kiwifruit powder were measured using commercial assay kits (Megazyme, Ireland). Sugar composition (sucrose, glucose, and fructose) was analyzed by high-performance anion-exchange chromatography with pulsed amperometric detection (HPAEC-PAD) following a modified method of Mellado-Mojica and López (2015). Kiwifruit powder (50 mg, db) was extracted with MilliQ water (10 mL) for 24 h at room temperature. The extract was then centrifuged (3000 × g, 15 min) and the supernatant filtered (0.22 μm) prior to analysis. The analysis was performed on a Dionex ICS 5000 system (Sunnyvale, CA, USA) equipped with a CarboPac PA-100 analytical column (4 × 250 mm) and a CarboPac PA100 guard column (4 × 50 mm) (Thermo Fisher Scientific, Waltham, MA, USA). The chromatographic separation was achieved using an isocratic mobile phase of 0.1 mol L^-1^ sodium hydroxide containing 0.2 mol L^-1^ sodium acetate at a flow rate of 0.5 mL min^-1^, with the column temperature maintained at 30°C and an injection volume of 25 μL. Sugars were quantified using external calibration curves. The chemical compositions of the powders were summarized in supplementary Table S1.

**Reference**

Mellado-Mojica E and Lopez MG, Identification, classification, and discrimination of agave syrups from natural sweeteners by infrared spectroscopy and HPAEC-PAD. Food Chem **167**: 349 – 357 (2015).

Supplementary Table S1 Chemical composition of pure kiwifruit powder and wheat flour

|  | Kiwifruit powder | Wheat flour |
| --- | --- | --- |
| Moisture (g kg^-1^) | 131 ± 1.13 | 126 ± 4.94 |
| Crude lipid (g kg^-1^) | 14.8 ± 0.517 | 18* |
| Protein (g kg^-1^) | 55.6 ± 0.231 | 134 ± 10.3 |
| Total starch (g kg^-1^) | 50.4 ± 4.86 | 771* |
| Amylose (g kg^-1^) | / | 198* |
| Total dietary fibre (g kg^-1^) | 160 ± 15.1 | 33* |
| Soluble dietary fibre (g kg^-1^) | 36.2 ± 3.00 | / |
| Insoluble fibre (g kg^-1^) | 123 ± 18.1 | / |
| Sugars |  | 27* |
| Glucose (g kg^-1^) | 238 ± 17.0 | / |
| Fructose (g kg^-1^) | 257 ± 14.0 | / |
| Sucrose (g kg^-1^) | n.d | / |
| Vitamin C (g kg^-1^) | 9.79 ± 0.689 | / |
| Ash (g kg^-1^) | 47.9 ± 8.81 | 31* |

Data were displayed as mean values ± standard deviation (n = 3); n.d, not detected; db, dry basis. *, the composition was reported by our previous study using the same sample (Cui & Zhu, 2020); /, not measured. The number of repetitions for the experiment is three.

Supplementary Table S2. Gel texture of kiwifruit flour and wheat flour mixtures after pasting analysis

| Samples | Hardness (N) | Adhesiveness | Springiness | Chewiness (N) |
| --- | --- | --- | --- | --- |
| KF-0 | 0.120 a | 1.139 a | 0.953 a | 0.197 a |
| KF-100 | 0.106 b | 1.024 a | 0.936 ab | 0.156 b |
| KF-200 | 0.072 c | 0.727 b | 0.898 b | 0.077 c |
| KF-300 | 0.052 d | 0.774 b | 0.839 c | 0.075 c |
| KF-400 | 0.036 e | 0.470 c | 0.773 d | 0.030 d |
| KF-500 | 0.025 e | 0.200 d | 0.699 e | 0.034 d |

KF, kiwifruit flour powder. KF-0 – 500, a range of flour mixtures where kiwifruit flour replaces 0 to 500 g kg^-1^ of wheat flour by weight. Different letters in the same column indicated significant difference (*p* < 0.05). The number of repetitions for the experiment is three.

Supplementary Table S3 Colour analysis of KCSB samples collected from processing steps

| Samples | | *L** | *a** | *b** |
| --- | --- | --- | --- | --- |
| KCSB-0 | Mixing | 92.14 a | -0.06 d | 9.56 e |
|  | Fermentation | 92.44 a | -0.08 c | 8.15 f |
|  | Proofing | 92.98 a | -0.10 b | 8.43 f |
|  | Steaming | 91.50 a | -0.35 e | 9.58 f |
|  | Storage | 92.87 a | -0.11 e | 8.16 e |
| KCSB-100 | Mixing | 92.34 a | -0.44 f | 10.01 e |
|  | Fermentation | 92.55 a | -0.61 e | 11.07 e |
|  | Proofing | 92.87 a | -0.51 c | 11.14 e |
|  | Steaming | 89.79 b | 0.37 d | 11.09 e |
|  | Storage | 89.63 b | 0.30 d | 11.12 c |
| KCSB-200 | Mixing | 89.06 b | 0.51 b | 12.96 d |
|  | Fermentation | 90.78 b | -0.45 d | 12.05 d |
|  | Proofing | 89.10 b | 0.95 a | 12.84 c |
|  | Steaming | 87.63 c | 1.08 c | 12.13 d |
|  | Storage | 89.95 b | 0.60 c | 11.01 d |
| KCSB-300 | Mixing | 88.04 b | -0.26 e | 14.60 c |
|  | Fermentation | 87.67 c | -0.12 c | 14.73 c |
|  | Proofing | 88.07 d | 0.83 a | 12.31 d |
|  | Steaming | 85.55 c | 1.38 b | 14.94 c |
|  | Storage | 87.74 c | 0.71 b | 12.63 b |
| KCSB-400 | Mixing | 85.60 c | 0.12 c | 16.16 b |
|  | Fermentation | 84.60 d | 0.12 b | 16.76 b |
|  | Proofing | 84.00 d | 0.72 a | 18.70 b |
|  | Steaming | 81.46 e | 1.46 b | 18.77 b |
|  | Storage | 80.33 d | 2.02 a | 19.37 a |
| KCSB-500 | Mixing | 83.02 d | 0.85 a | 19.68 a |
|  | Fermentation | 81.79 e | 0.69 a | 20.75 a |
|  | Proofing | 82.24 e | 0.88 a | 20.10 a |
|  | Steaming | 77.08 f | 2.67 a | 22.42 a |
|  | Storage | 80.31 d | 1.95 a | 19.35 a |

*L**, the lightness; *a**, the redness-greenness; *b**, the yellowness-blueness; KCSB-0 – 500, the Chinese steamed bread made with wheat flour partially replaced by kiwifruit powder (0 – 500 g kg^-1^). Different letters in the same column and of the same CSB indicated significant difference (*p* < 0.05).

Supplementary Table S4 Colour analysis of fresh KCSB samples

| Sample | Crust | | | | Crumb | | | | |
| --- | --- | --- | --- | --- | --- | --- | --- | --- | --- |
|  | *L** | *a** | *b** | Whiteness Index |  | *L** | *a** | *b** | Whiteness Index |
| KCSB-0 | 89.52 a | -0.14 e | 22.47 a | 75.21 a |  | 89.23 a | -0.14 d | 20.97 a | 23.58 c |
| KCSB-100 | 87.58 b | 1.65 d | 21.61 a | 75.02 a |  | 87.67 b | 1.00 c | 20.16 ab | 23.66 c |
| KCSB-200 | 71.04 c | 2.16 c | 18.73 b | 65.44 b |  | 84.05 c | 1.16 c | 19.48 bc | 25.21 b |
| KCSB-300 | 66.81 d | 4.49 b | 16.86 c | 62.50 c |  | 73.86 d | 2.35 b | 19.09 c | 32.46 a |
| KCSB-400 | 63.10 e | 4.84 ab | 15.59 d | 59.65 d |  | 73.22 d | 3.21 a | 18.07 d | 32.48 a |
| KCSB-500 | 59.35 f | 5.04 a | 13.18 e | 56.97 e |  | 72.80 e | 3.63 a | 17.13 e | 32.35 a |

*L**, the lightness; *a**, the redness-greenness; *b**, the yellowness-blueness; Whiteness index, the degree of whiteness. KCSB-0 – 500, the Chinese steamed bread made with wheat flour partially replaced by kiwifruit powder (0 – 500 g kg^-1^). Different letters (a-f) in the same column indicated significant difference (*p* < 0.05).

Table S5 Microbiological shelf life of fresh KCSB during seven-day storage


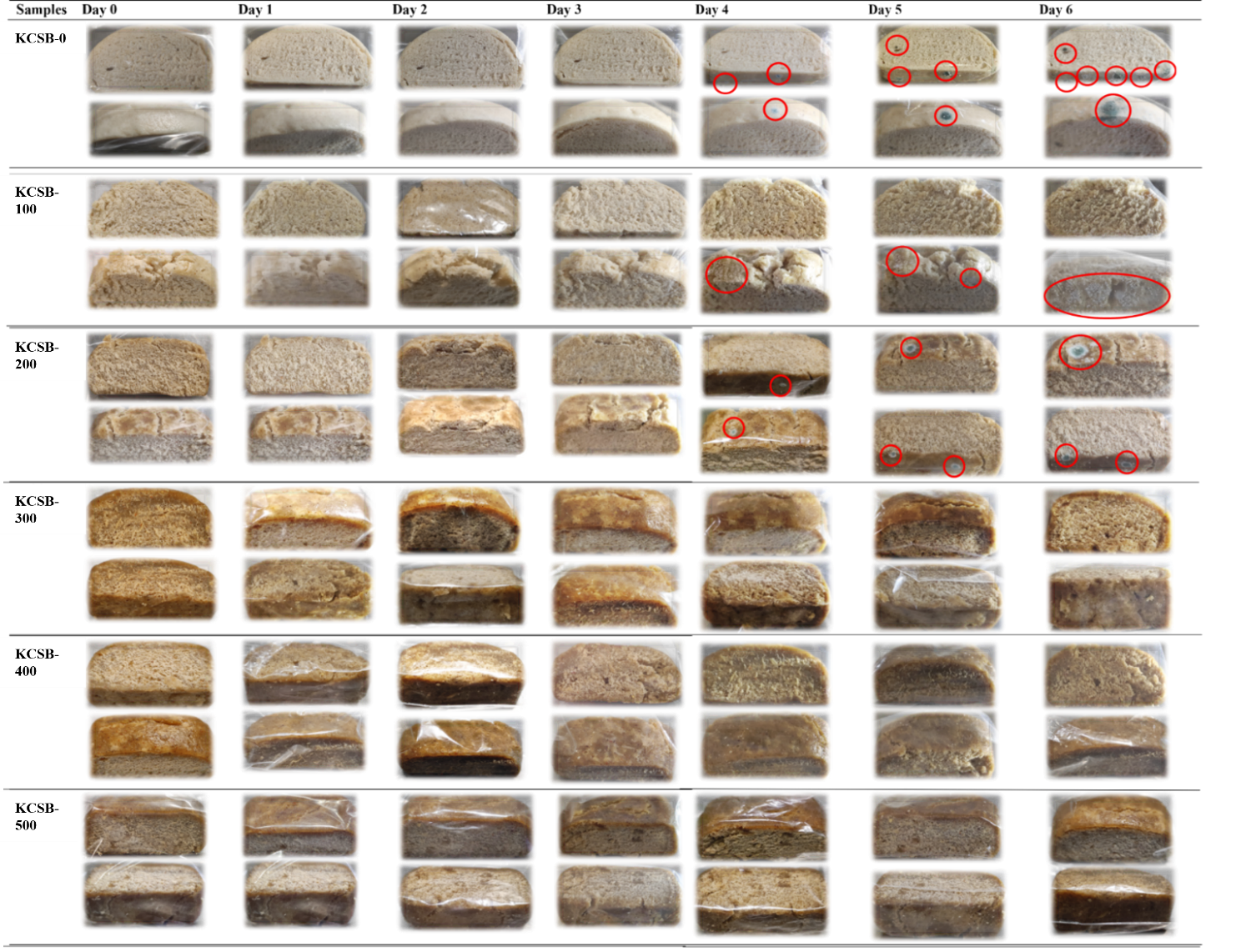


KCSB-0 – 500, the Chinese steamed bread made with wheat flour partially replaced by kiwifruit powder (0 – 500 g kg^-1^). Red circles demonstrated the emerging mould on the samples


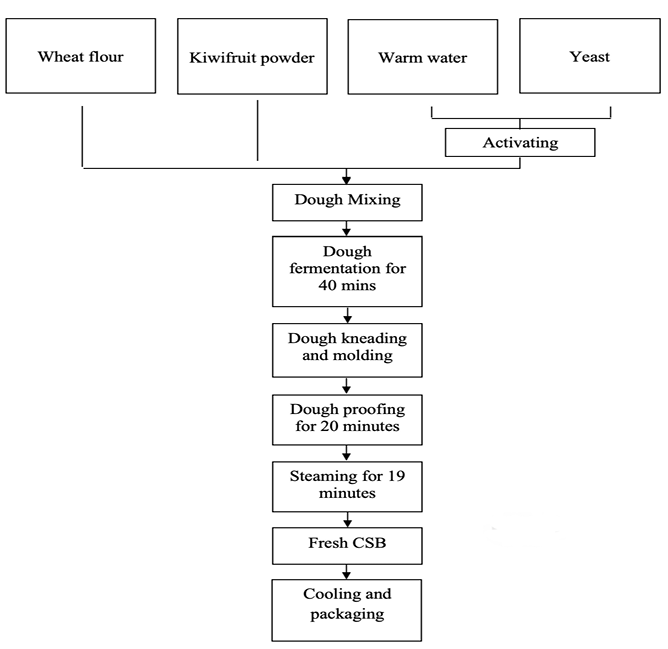


Supplementary Fig. S1 Diagram of breadmaking process
